# Microwave Staring Correlated Imaging Method Based on Steady Radiation Fields Sequence

**DOI:** 10.3390/s20236859

**Published:** 2020-11-30

**Authors:** Jianlin Zhang, Bo Yuan, Zheng Jiang, Yuanyue Guo, Dongjin Wang

**Affiliations:** Key Laboratory of Electromagnetic Space Information, Chinese Academy of Sciences, University of Science and Technology of China, Hefei 230026, China; yuanb@mail.ustc.edu.cn (B.Y.); jiangz10@mail.ustc.edu.cn (Z.J.); yuanyueg@ustc.edu.cn (Y.G.); wangdj@ustc.edu.cn (D.W.)

**Keywords:** microwave staring correlated imaging, steady radiation fields sequence, coherent excitations, optimized source design

## Abstract

Microwave Staring Correlated Imaging (MSCI) is a newly proposed computational high-resolution imaging technique. The imaging performance of MSCI with the existence of modeling errors depends on the properties of the imaging matrix and the relative perturbation error resulted from existing errors. In conventional transient-radiation-fields-based MSCI, which is commonly accomplished by utilizing random frequency-hopping (FH) waveforms, the multiple transmitters should be controlled individually and simultaneously. System complexity and control difficulty are hence increased, and various types of modeling errors are introduced as well. The computation accuracy of radiation fields is heavily worsened by the modeling errors, and the transient effect makes it hard to take direct and high-precision measurements of the radiation fields and calibrate the modeling errors with the measuring result. To simplify the system complexity and reduce error sources, in this paper, steady-radiation-fields-sequence-based MSCI (SRFS-MSCI) method is proposed. The multiple transmitters are excited with coherent signals at the same observation moment, with the signal frequency varying in the whole frequency band during the imaging process. By elaborately designing the array configuration and the amplitude and phase sequences of the coherent transmitters, the SRFS-MSCI is thus implemented. Comparing the system architecture of the proposed SRFS-MSCI with the conventional random FH-based MSCI, it can be found that the proposed method significantly reduces the number of baseband modules and simplifies the system architecture and control logic, which contributes to reducing error sources such as baseband synchronization errors and decreasing deterioration caused by error cascade. To further optimize the design parameters in the proposed SRFS-MSCI system, the Simulated Annealing (SA) algorithm is utilized to optimize the amplitude sequences, the phase sequences, and the antenna positions individually and jointly. Numerical imaging experiments and real-world imaging experiment demonstrate the effectiveness of the proposed SRFS-MSCI method that recognizable high-resolution recovery results are obtained with simplified system structure and optimized system parameters.

## 1. Introduction

Radar imaging technology has attracted increasing attention since the 1960s for its ability to work all day and in all weather environments [1,2,3,4,5]. Among the multiple kinds of radar imaging applications, there exists an important demand for radar staring imaging, i.e., continuous observation of a fixed area such as for key area surveillance, disaster monitoring, etc. However, conventional staring imaging radar such as real aperture radar (RAR) cannot obtain satisfying high-resolution results, because the azimuth resolution of RAR is limited by the aperture size of the transmitting antennas [6,7]. To achieve higher resolution in staring imaging applications, Microwave Staring Correlated Imaging (MSCI) was recently proposed and investigated [8,9], inspired by optical Ghost Imaging (GI) [10,11]. The imaging problem of MSCI is often formed as a linear inverse problem, where the radiation fields are precomputed according to radar system parameters. Learning from the random features of the thermal light source in GI, the essential principle of MSCI is to construct temporal–spatial stochastic radiation fields (TSSRF), which possess stochastic characteristics both in spatial differentiation and time variation. By the Correlation Process (CP) of TSSRF and the corresponding scattering echo, targets within the imaging region can be reconstructed with high imaging resolution. MSCI achieves superior imaging performance without the relative motion between radar and target, and has thus attracted increasing attention and obtained rapid developments in many aspects, such as imaging algorithms [12,13,14], outfield experiments [15], imaging based on unsteady aerostat platform [16,17], etc.

**Figure 1:**
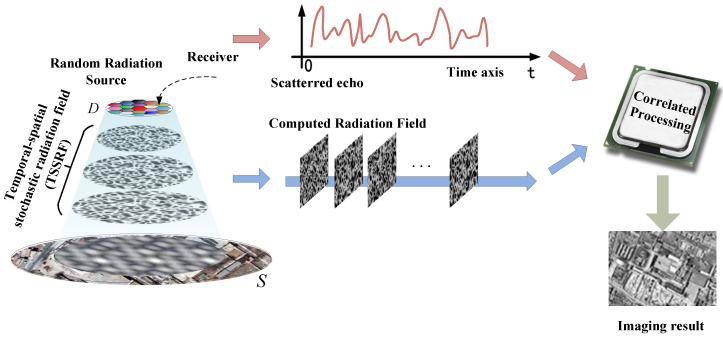
Procedure of Microwave Staring Correlated Imaging (MSCI).

The detailed process of MSCI is illustrated in Figure 1. The imaging resolution of MSCI is determined by the stochastic characteristics of TSSRF, or the property of the imaging matrix, which is the temporal-spatial discretization of the TSSRF. Patterns of radiation fields, i.e., different rows and columns of the imaging matrix, should be linearly independent. Under such circumstances, neighboring scattering targets within the antenna beam are illuminated by the diverse patterns of radiation fields, and the corresponding scattering echoes at different positions in the imaging region possess diverse temporal characteristics, providing the possibility of targets decoupling [15,18,19].

To implement the needed TSSRF in the region of interest, efforts have been made on the generation and optimization of the TSSRF or the imaging matrix. Considering requirements of remote sensing and engineering limitations such as bandwidth limitation and power limitation, multitransmitter architecture with simultaneously emitted randomly modulated waveforms is most commonly utilized in MSCI [9,19,20,21]. Among the different random modulations, random Frequency Hopping (FH) signals are most commonly employed because of the constant-envelope feature and the frequency agility [15,22,23]. An optimization algorithm was adopted to acquire a well-behaved frequency code design of the FH signals based on minimizing the condition number of the sensing matrix [24]. Temporal–Spatial Distribution Entropy (TSDE) was proposed in [25] as the optimization target of the joint design of waveform parameters and array configuration. A novel bistatic MSCI architecture was presented in [26] to observe the targets from larger aspect angles and to form TSSRF with better stochastic feature. Arrays with special electronic materials have also been investigated for example, a reflective metasurface was used in MSCI to achieve the random radiation fields [27]. An orthogonal radiation field construction method was proposed based on approximating the radiation field to a set of two-dimensional orthogonal basis functions [28]. Owing to the abundant designs of MSCI radiation source, TSSRF are generated in the imaging region and targets are well-reconstructed with high resolution.

However, multiple transmitters and the simultaneous emitting scheme of randomly modulated waveforms of each transmitter result in high system complexity and high control difficulty. The waveform parameters of each transmitter are independent with each other, which means separate transmitting devices and individual controls are needed, especially modules referring to frequency synthesizing such as the baseband device and mixer. Meanwhile, randomly modulated waveforms of multiple transmitters lead to the instantaneous effect of the radiation fields, i.e., the TSSRF are independent in spatial distribution but transient in time domain. As a high-resolution imaging technique, MSCI requires precise information of the TSSRF. Unlike thermal light fields in optical GI, where a splitter and charge-coupled device (CCD) are used to directly record the accurate actual temporal-spatial information of the detecting light beam, the bucket receiving effect of microwave antenna makes it hard to directly and precisely record the whole microwave TSSRF with high spatial precision. Therefore, the radiation fields need to be computed according to the predesigned transmitting parameters and propagation procedure. Hence, the estimation precision of echo sampling delays and the radar system errors from device imperfections significantly influence the computation accuracy of TSSRF.

On the one hand, the transient effect of TSSRF in MSCI will increase the mismatch between computed TSSRF and echo samples once the propagation time delays are not well estimated, resulting in failure of target reconstruction. Meanwhile, the instability of transient radiation fields makes it more likely for field measurement calibration to go wrong under the demand of high spatial precision, which means difficulty in correcting the computed results of TSSRF with actual measurement references. On the other hand, errors always exist in real radar systems. In the abovementioned transient-fields-based MSCI system, multiple transmitting channels are needed to individually generate waveforms of different frequencies, which requires individual devices and separate controls, especially baseband device and mixers. The complex system architecture and control logic bring in coexisting system errors, such as gain-phase error, synchronization error, array position error, timing mismatch between radiation field and scattering echo, etc. Although methods have been proposed to solve the imaging problems of MSCI with errors, they both have some limitations. For example, sparsity-driven self-calibration methods were proposed to compensate the abovementioned errors by iteratively estimating the modeling errors and reconstructing the imaging result [29,30,31], but the method is only suitable for sparse imaging targets and the error calibration ranges are limited by algorithm convergence. In [32], a reference receiver was added in the imaging system to directly estimate the the gain-phase errors and synchronization errors by analyzing the acquisition of the direct transmitting signals. The method requires high Signal-to-Noise Ratio (SNR) of the direct signals to ensure the accuracy of error estimates, but may not be guaranteed in real radar systems due to antenna sidelobes and shieldings between transmitting antennas. Thus, the transient radiation-fields-based MSCI can achieve high imaging resolution, whereas high system complexity is meanwhile introduced with increasing possibility of unstable imaging results and reconstruction failure.

Focusing on the abovementioned defects, a steady-radiation-fields-sequence-based MSCI (SRFS-MSCI) is proposed. Different from transient-radiation-fields-based MSCI, the multiple transmitters of SRFS-MSCI are excited by coherent signals with diverse amplitudes and phases. Herein, the diversity of radiation fields patterns are guaranteed by different amplitude-phase sequences at varying frequencies to achieve high imaging resolution; meanwhile, in the radiation fields, the power distribution in the imaging region is steady under the same parameter scheme, which is beneficial for taking direct measurements of the radiation fields and estimating the modeling errors. In this case, individual transmitting channels, especially individual baseband devices and mixers, are replaced by power dividers, which significantly reduces the system complexity and the sources of the modeling errors. To further optimize the waveform and array configuration in the case of steady radiation fields sequence, the Simulated Annealing (SA) algorithm is utilized to optimize the amplitude sequences, the phase sequences, and the antenna positions individually and jointly. Numerical experiments and real-world experiments demonstrate the effectiveness of the proposed method that recognizable high-resolution result is obtained with simplified system structure.

The rest of the paper is organized as follows. The general formulation of MSCI is derived in Section 2. The imaging performance is obtained based on matrix perturbation theory, and the influence of different imaging errors are analyzed from numerical imaging experiments with random frequency-hopping waveforms. In Section 3, the system design of SRFS-MSCI is proposed and compared with conventional transient-radiation-fields-based MSCI, and the Simulated Annealing (SA) algorithm is utilized in waveform optimization method based on minimizing the condition number of the imaging matrix. In Section 4, numerical imaging experiments and a real-world imaging experiment are conducted to verify the effectiveness of the proposed SRFS-MSCI scheme. Finally, the paper is concluded in Section 5.

## 2. Formulation of MSCI and the Defects Caused by Errors

### 2.1. Formulation of MSCI

**Figure 2:**
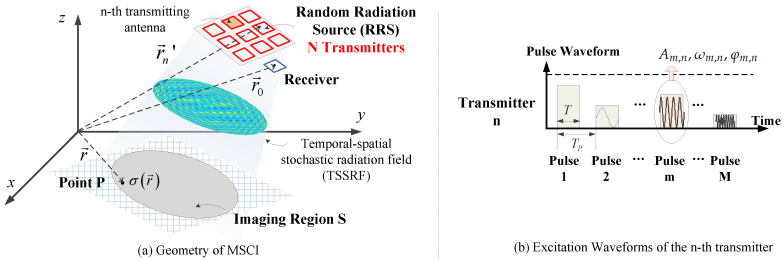
The geometry of MSCI and the excitation waveform.

A typical geometry of MSCI including *N* transmitters and a single receiver is illustrated in Figure 2a. The transmitting antennas are within a planar array located on a stationary platform. *P* is an arbitrary point in the 2-D imaging region *S*. ${\vec{r}}_{0}$, ${\vec{r}}_{n}^{\prime }$, and $\vec{r}$ are the position vectors of the receiver, the *n*-th transmitting antenna $D_{n}$, and the arbitrary point *P*, respectively.

The imaging system generates *M* pulses by simultaneous transmission of all the N transmitters [33]. The excitation signal to the *n*-th transmitting antenna has the following form as multiple pulse signal, as shown in Figure 2b.

(1) $$s_{m,n}\left(t\right)=rect\left(\frac{t-mT_{p}}{T}\right)A_{m,n}e^{j(\omega_{m,n}\left(t-mT_{p}\right)+\varphi_{m,n})},m=1,2,\ldots ,M,n=1,2,\ldots ,N,$$

where rect(t) is the rectangular window function, *M* is the number of pulses, $T_{p}$ is the pulse repetion interval, and *T* is the pulse width. $A_{m,n},\omega_{m,n},\varphi_{m,n}$ are the amplitude, frequency, and phase of the excitation signal to the *n*-th transmitting antenna in the *m*-th pulse, respectively. Based on the Huygens’ principle, the radiation field on the imaging region *S* can be derived from wave equation as

(2) $$E^{rad}\left(\vec{r},t\right)=\sum_{n=1}^{N}\frac{F_{n}\left({\vec{r}}_{n}^{\prime },\vec{r},\omega \right)\cdot s_{n}\left(t-\frac{|\vec{r}-{\vec{r}}_{n}^{\prime }|}{c}\right)}{4\pi |\vec{r}-{\vec{r}}_{n}^{\prime }|},$$

where $F_{n}\left({\vec{r}}_{n}^{\prime },\vec{r},\omega \right)$ denotes the radiation pattern of the *n*-th antenna under frequency ω, and *c* is the speed of light. Herein, an assumption is made that the mutual coupling between neighboring antennas is ignored.

Let $\sigma (\vec{r})$ denote the backscattering coefficient of the target in *S*. With the existence of additive noise, the received echo can be expressed as

(3) $$E^{sca}\left({\vec{r}}_{0},t\right)={\int \int }_{S}E^{eff}\left(\vec{r},{\vec{r}}_{0},t\right)\cdot \sigma \left(\vec{r}\right)d{\vec{r}}^{2}+n\left(t\right),$$

where the modified radiation field is defined as

(4) $$E^{eff}\left(\vec{r},{\vec{r}}_{0},t\right)=\frac{1}{4\pi |\vec{r}-{\vec{r}}_{0}|}E^{rad}\left(\vec{r},t-\frac{\left|\vec{r}-{\vec{r}}_{0}\right|}{c}\right)=\sum_{n=1}^{N}\frac{F_{n}\left({\vec{r}}_{n}^{\prime },\vec{r}\right)\cdot s_{m,n}\left(t-\frac{\left|\vec{r}-{\vec{r}}_{n}^{\prime }|+|\vec{r}-{\vec{r}}_{0}\right|}{c}\right)}{16\pi^{2}|\vec{r}-{\vec{r}}_{n}^{\prime }||\vec{r}-{\vec{r}}_{0}|}.$$

Equation (3) is the imaging equation in form of Fredholm integral equation of the first kind, and is a linear inverse problem. To solve the inverse imaging problem by numerical computation, the continuous imaging area *S* is divided into $L=L_{x}\times L_{y}$ discrete imaging grid-cells according to the required imaging resolution. The scattering echoes are sampled from the *M* pulses in time domain, with each pulse providing one echo sample and the corresponding radiation field. Thus, the integral imaging Equation (3) is discrete as the matrix-form

(5) $$\vec{y}=E\cdot \vec{\sigma }+\vec{n},$$

where $\vec{y}={\left[E^{sca}\left({\vec{r}}_{0},t_{1}\right),E^{sca}\left({\vec{r}}_{0},t_{2}\right),\ldots ,E^{sca}\left({\vec{r}}_{0},t_{M}\right)\right]}^{T}$ denotes the echo vector, $\vec{\sigma }={\left[\sigma \left({\vec{r}}_{1}\right),\sigma \left({\vec{r}}_{2}\right),\ldots ,\sigma \left({\vec{r}}_{L}\right)\right]}^{T}$ is the scattering coefficient vector, and $\vec{n}={\left[n\left(t_{1}\right),n\left(t_{2}\right),\ldots ,n\left(t_{M}\right)\right]}^{T}$ is the additive noise vector. E is the M×L imaging matrix or sensing matrix

(6) $$\begin{array}{c} E=\left[\begin{array}{cccc} E^{eff}\left({\vec{r}}_{1},t_{1};{\vec{r}}_{0}\right) & E^{eff}\left({\vec{r}}_{2},t_{1};{\vec{r}}_{0}\right) & \ldots & E^{eff}\left({\vec{r}}_{L},t_{1};{\vec{r}}_{0}\right)\\ E^{eff}\left({\vec{r}}_{1},t_{2};{\vec{r}}_{0}\right) & E^{eff}\left({\vec{r}}_{2},t_{2};{\vec{r}}_{0}\right) & \ldots & E^{eff}\left({\vec{r}}_{L},t_{2};{\vec{r}}_{0}\right)\\ \vdots & \vdots & \vdots \\ E^{eff}\left({\vec{r}}_{1},t_{M};{\vec{r}}_{0}\right) & E^{eff}\left({\vec{r}}_{2},t_{M};{\vec{r}}_{0}\right) & \ldots & E^{eff}\left({\vec{r}}_{L},t_{M};{\vec{r}}_{0}\right) \end{array}\right]. \end{array}$$

Target reconstruction can be achieved by solving (5) with various correlation algorithms. For example, based on the objective of minimizing imaging error, the scattering coefficient $\vec{\sigma }$ can be recovered with Least Square (LS) method, described as

(7) $$\tilde{\vec{\sigma }}=\underset{\vec{\sigma }}{\arg\min}\left\{∥\vec{y}-E\cdot \vec{\sigma }{∥}_{2}^{2}\right\},$$

where ${∥\vec{a}∥}_{2}$ denotes the $L_{2}$-norm of the vector $\vec{a}$.

### 2.2. Imaging Performance of MSCI Based on Matrix Perturbation Theory

To ensure the solution of the matrix imaging equation, each equation of the equation set should be independent, which can be evaluated by the first-order normalized correlation coefficient as

(8) $$R\left(t_{1},t_{2}\right)=\frac{\langle \vec{E}\left(t_{1}\right),\vec{E}\left(t_{2}\right)\rangle }{{∥\vec{E}\left(t_{1}\right)∥}_{2}\cdot {∥\vec{E}\left(t_{2}\right)∥}_{2}},$$

where $\vec{E}\left(t_{m}\right)$ is the *m*-th row vector from the imaging matrix E, $<\;\vec{a},\vec{b}\;>$ denotes inner product. The more independent radiation fields generated, the higher imaging performance MSCI achieves.

In a noise-free situation, a full-column rank imaging matrix E contributes to accurate reconstruction of $\vec{\sigma }$, i.e., rank(E)=L, which potentially requires M≥L. The target can be accurately reconstructed under a noise-free condition, meaning the resolution of MSCI achieves the grid size in the imaging region *S*.

Then, under noise condition, the imaging performance measured by relative imaging error (RIE) satisfies the following inequality according to norm compatibility as

(9) $$RIE=\frac{∥\Delta \vec{\sigma }∥}{∥\vec{\sigma }∥}=\frac{∥\tilde{\vec{\sigma }}-\vec{\sigma }∥}{∥\vec{\sigma }∥}\leq \;\left\{\begin{array}{cc} cond\left(E^{H}E\right)\cdot \frac{∥\vec{n}∥}{∥\vec{y}-\vec{n}∥}, & M\;>\;L\\ cond\left(E\right)\cdot \frac{∥\vec{n}∥}{∥\vec{y}-\vec{n}∥}, & M\;=\;L \end{array}\right.$$

where cond(E) is the condition number of E, $∥\vec{\sigma }∥$ is $l_{2}$-norm of vector $\vec{\sigma }$, and ${∥\vec{y}\;-\;\vec{n}∥}^{2}/{∥\vec{n}∥}^{2}$ approximately equals to SNR.

Consider the imaging performance defected by the modeling errors in MSCI under noise-free condition. The effect resulted from modeling errors can be concluded as an additive perturbation matrix to the imaging matrix, described as

(10) $${\vec{E}}^{sca}=\left(E+\Delta E\right)\cdot \left(\vec{\sigma }+\Delta \vec{\sigma }\right).$$

Then, based on matrix perturbation theory, the RIE satisfies

(11) $$RIE=\frac{∥\Delta \vec{\sigma }∥}{∥\vec{\sigma }∥}=\frac{∥\tilde{\vec{\sigma }}-\vec{\sigma }∥}{∥\vec{\sigma }∥}\leq \;\left\{\begin{array}{cc} cond\left(E^{H}E\right)\cdot \frac{∥\Delta E∥}{∥E∥}, & M\;>\;L\\ cond\left(E\right)\cdot \frac{∥\Delta E∥}{∥E∥}, & M\;=\;L \end{array}\right.$$

where $∥E∥$ is the spectral norm of the matrix E.

The relative perturbation error (RPE) $∥\Delta E∥/∥E∥$ added to the imaging matrix is entirely caused by the modeling errors. As the types of modeling errors and their corresponding error levels increase, the RPE of the imaging matrix gets larger. Therefore, the imaging performance of MSCI with existence of modeling errors mainly depends on two aspects: the condition number of the imaging matrix, and the levels of the modeling errors.

### 2.3. Defects in MSCI with Random Frequency-Hopping Waveforms

**Figure 3:**
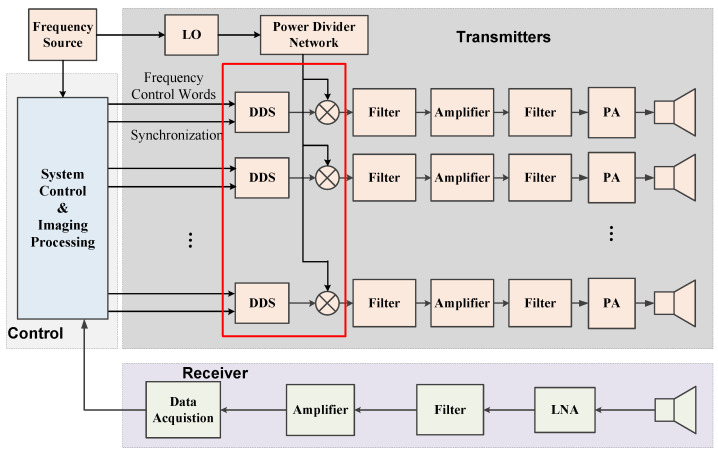
A typical system diagram of conventional MSCI emitting random frequency-hopping waveforms. LO—Local Oscillator; DDS—Direct Digital Synthesis; PA—Power Amplifier; LNA—Low Noise Amplifier.

In consideration of the constant envelope characteristic and the frequency agility of frequency-hopping waveforms, the conventional transient-fields-based MSCI utilizes random frequency-hopping signals as the radar waveforms. A typical radar system diagram of MSCI based on random frequency-hopping signals is illustrated in Figure 3. As can be seen, the multiple transmitters work under the unified clock source, but the excitations of the multiple channels are signals at different frequencies and are generated by the mixing of individual Local Oscillator (LO) and Direct Digital Synthesis (DDS). The LO’s of multiple channels are generated after a power divider, and the DDS of each channel is individually controlled by the predetermined frequency control words. Thus, the excitations of different channels are incoherent. The transmitting array consisting of *N* transmitters requires the number of *N* separate DDS and *N* related mixers.

**Figure 4:**
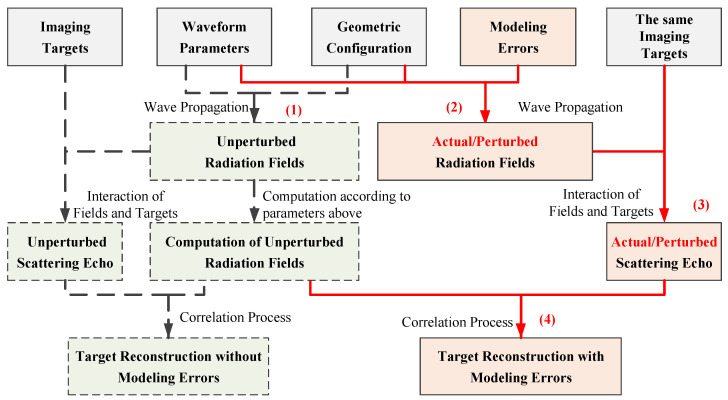
Imaging procedure of MSCI with modeling errors.

As a high-resolution computational imaging technique, the correlation process of MSCI requires accurate computation of the radiation fields according to the predesigned system parameters, and strict match between the actual scattering echo and the computed radiation fields. However, due to the transient property of the radiation fields with the usage of random FH waveform, the computational accuracy of the radiation fields is sensitive to inevitable modeling errors of actual radar systems. Slight perturbations of the system parameters lead to evident variations of the computed radiation field to its actual value. Worse still, multiple kinds of modeling errors usually coexist in the system, which brings in further disadvantages to the imaging performance. The detailed procedures of MSCI with modeling errors are illustrated in Figure 4. The subdiagram with a dash line on the left side shows the procedure of MSCI without modeling errors; while the subdiagram with full line on the right side demonstrates MSCI with modeling errors. As can be seen, the modeling errors directly influence the actual situation of radiation fields and the actual scattering echoes; while the correlation process utilizes the computation result of unperturbed radiation fields because of the lack of prior information about the modeling errors, for example, the information about existence, types, and magnitudes of the modeling errors. Therefore, there exists a mismatch between the perturbed scattering echo and unperturbed computed radiation fields, resulting in reduction of imaging performance. Simulations are conducted to visually demonstrate the individual defect of modeling errors according to Figure 4 in software MATLAB R2019b for academic use. The detailed simulation procedures are listed below.

(1) Simulation and computation of unperturbed radiation fields without modeling errors: With the predesigns of the waveform parameters and geometric configuration, simulate the unperturbed radiation fields according to wave propagation procedure and obtain computation results of unperturbed radiation fields according to (4).
(2) Simulation of actual radiation fields with the existence of modeling errors: According to the prior information about modeling errors, obtain actual values of the waveform parameters and geometric configuration, and make revisions on (1) and (2). Then, recompute the radiation fields according to wave propagation procedure according to (4) with the revised (1) and (2).
(3) Simulation of actual scattering echoes with the existence of modeling errors: With the interaction of radiation fields and the given imaging target, the actual scattering echoes are obtained according to (3).
(4) Correlation process of MSCI with modeling errors: Form the imaging equation in matrix form (5) with the computed radiation fields in Step (1) and the actual scattering echo in Step (3), and reconstruct the imaging target with the relative imaging algorithm (7).
(5) Change the types and magnitudes of the modeling errors and obtain corresponding imaging results to demonstrate the detailed influences of diverse kinds of modeling errors.

There exists a potential modeling error where the calculated radiation field mismatches the selected echo sample, resulting in a failure of target reconstruction. As can be seen in (5), to ensure the matching of echo signals and the radiation fields, the TSSRF should be computed strictly according to the sampling time $t_{1},t_{2},\ldots ,t_{M}$ of the echo signals and the propagation procedure. Once the echo sampling times mismatch the computation times, which can be easily caused by imperfections of devices, a wrong imaging equation is established as follows when ignoring the additive noise:

(12) $$\begin{array}{c} \left[\begin{array}{c} E^{sca}\left({\vec{r}}_{0},t_{1}\right)\\ E^{sca}\left({\vec{r}}_{0},t_{2}\right)\\ \vdots \\ E^{sca}\left({\vec{r}}_{0},t_{M}\right) \end{array}\right]=\left[\begin{array}{cccc} E^{eff}\left({\vec{r}}_{1},t_{1}^{\prime };{\vec{r}}_{0}\right) & E^{eff}\left({\vec{r}}_{2},t_{1}^{\prime };{\vec{r}}_{0}\right) & \ldots & E^{eff}\left({\vec{r}}_{L},t_{1}^{\prime };{\vec{r}}_{0}\right)\\ E^{eff}\left({\vec{r}}_{1},t_{2}^{\prime };{\vec{r}}_{0}\right) & E^{eff}\left({\vec{r}}_{2},t_{2}^{\prime };{\vec{r}}_{0}\right) & \ldots & E^{eff}\left({\vec{r}}_{L},t_{2}^{\prime };{\vec{r}}_{0}\right)\\ \vdots & \vdots & \vdots \\ E^{eff}\left({\vec{r}}_{1},t_{M}^{\prime };{\vec{r}}_{0}\right) & E^{eff}\left({\vec{r}}_{2},t_{M}^{\prime };{\vec{r}}_{0}\right) & \ldots & E^{eff}\left({\vec{r}}_{L},t_{M}^{\prime };{\vec{r}}_{0}\right) \end{array}\right]\cdot \left[\begin{array}{c} \sigma ({\vec{r}}_{1})\\ \sigma ({\vec{r}}_{2})\\ \ldots \\ \sigma ({\vec{r}}_{L}) \end{array}\right]. \end{array}$$

The imaging matrix E(t) is perturbed as $E(t^{\prime })$. Considering that the radiation fields of conventional MSCI is transient in time domain, the perturbation degree is getting worse in general as the timing mismatch increases, while some periodic phenomenon may also exist in local areas associated with period time of electromagnetic waves. Hence, the imaging performance follows the similar changing pattern. Meanwhile, the transient effect makes it difficult to calibrate the computed radiation fields by measurements because the radiation fields change rapidly after a relatively short time duration.

**Figure 5:**
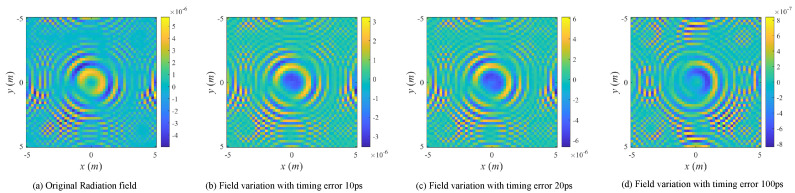
Original radiation field and field variations versus different types of imaging errors.

**Figure 6:**
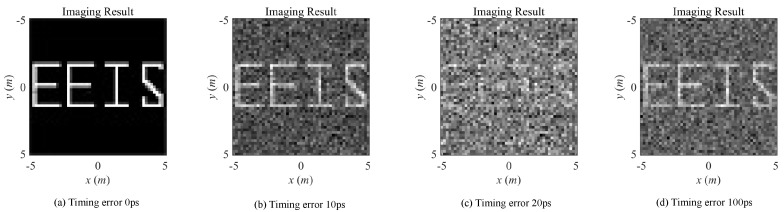
Imaging results with different levels of timing mismatch errors.

**Figure 7:**
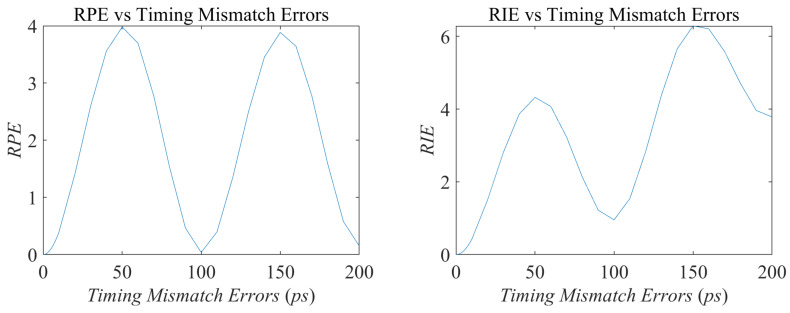
Relative perturbation error (RPE) and relative imaging error (RIE) versus timing mismatch errors.

With the simulation parameters listed in Table 1, the radiation fields and the field variations with the existence of different time mismatches are listed in Figure 5, and imaging results under noise-free condition are illustrated in Figure 6. The RPE and RIE are demonstrated in Figure 7. As can be seen in Figure 6 and Figure 7b, with the increasing timing mismatch, the imaging results get worse in general, but there exist potential periodic changes of RPE and RIE with relation to the electromagnetic cycle 100 ps at center frequency 10 GHz.

As for other modeling errors, with the abovementioned architecture design of the MSCI system, multiple kinds of modeling errors are brought into the imaging system. The multichannel synchronization errors, multichannel consistency errors, and gain-phase errors are brought into the system by the following modules: the power divider, DDS, the mixer, and the filter, etc. Different modules bring in different error characteristics and different error levels. The coexisting of these modeling errors and the cascade of the system modules exacerbate the modeling errors and make it hard to directly measure the modeling errors by system calibration, especially when the multiple transmitters work at different frequencies and the radiation fields are transient in time domain. The measurement requires special designs and relatively high precision.

**Figure 8:**
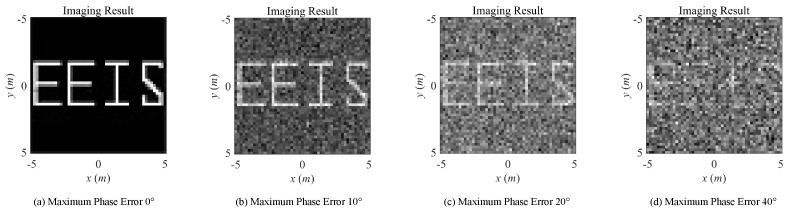
Imaging results with different levels of phase errors.

**Figure 9:**
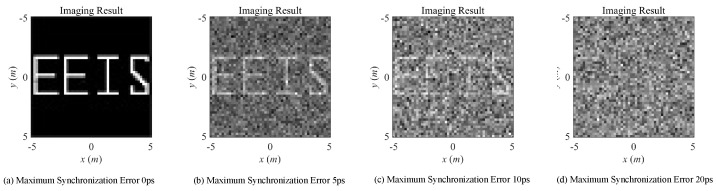
Imaging results with different levels of synchronization errors.

**Figure 10:**
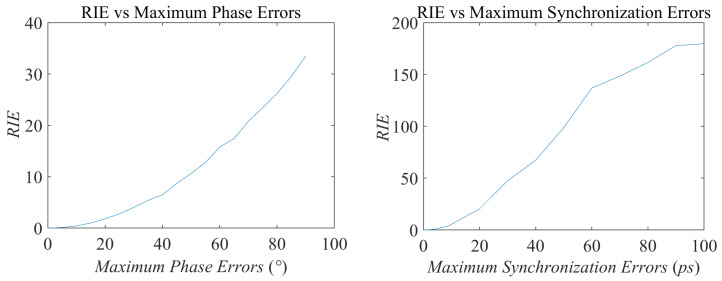
RIE versus different types of imaging errors.

Under the same simulation scenario listed in Table 1, the imaging results and RIE are illustrated as follows with the existence of modeling errors. The imaging results under noise-free condition of conventional transient-radiation-fields-based MSCI under different levels of phase errors, synchronization errors, and timing mismatch are demonstrated in Figure 8 and Figure 9, respectively. The corresponding RIEs are shown in Figure 10. As can be seen, slight modeling errors result in deteriorating imaging results. In actual radar systems, the necessarily existing noise will further worsen the imaging performance. Even under noise-free conditions, the 40° level of phase errors and 10 ps level synchronization errors, under which the recognizable target reconstruction results are obtained, put forward a strict demand to system design and physical implementation, especially considering that the signal cycle of X-band microwave field is about 100 ps. As can be predicted, the imaging results will get worse when the errors are coexisting, mutually cascaded, and coupled.

## 3. MSCI Based on Steady Radiation Fields Sequence

In the abovementioned conventional MSCI, the transient effect of the radiation fields leads to sensitivity to imaging errors, especially time errors and phase errors, and leads to difficulty in calibration by measurement as well. Considering that the transient effect of TSSRF are resulted from different excitation frequencies, especially that the radiation field within a pulse under a determined frequency set is transient itself, it is necessary to investigate whether there is a way to generate feasible TSSRF with coherent excitations. In a coherent excitation situation, the spatial distribution of the radiation fields maintain similar structure within the same set of parameters, but differ among different pulses, which means a smoother temporal transient effect than the abovementioned conventional MSCI. According to (1) and (4), the radiation fields are relative with antenna radiation patterns; transmitting antenna positions; and parameters of excitations including amplitudes, frequencies, and phases. With the lack of frequency design, the amplitudes and phases of excitations and antenna positions can be utilized to generate the needed radiation fields. Having the same frequency of multiple transmitters leads to coherent excitations and a steady radiation field, especially if the power distribution in the imaging region remains unchanged. The random features of TSSRF are guaranteed by varying parameters of amplitudes and phases of multiple transmitters during different pulses, i.e., a steady radiation fields sequence.

### 3.1. System Design of Steady-Radiation-Fields-Sequence-Based MSCI

**Figure 11:**
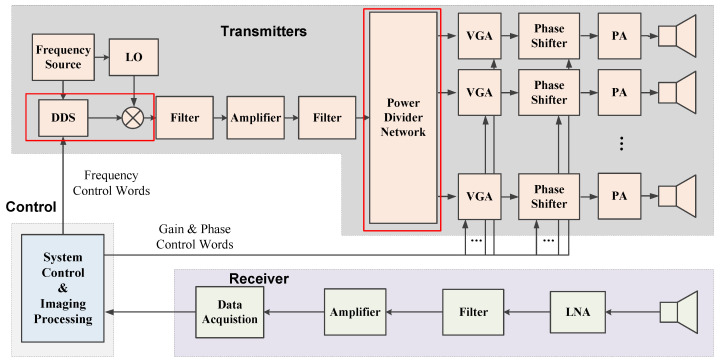
System architecture of SRFS-MSCI.

The system of the steady-radiation-fields-sequence-based MSCI is illustrated in Figure 11. Considering that the excitation signals of the multiple transmitters are coherent, i.e., the same frequency, a power divider network is utilized instead of multiple and individual transmitting channels. With *N* transmitting antennas in the imaging system, conventional MSCI system includes *N* DDS and *N* mixers, while the number of DDS and mixer in the proposed SRFS-MSCI system is reduced to 1, as compared in the red rectangle in Figure 3 and Figure 11. Considering that the synchronization errors mainly come from the frequency synthesizing procedure, the proposed method can significantly reduce the time errors. The essential design of the SRFS-MSCI is the amplitude and phase designs of the excitation signals, thus, the Variable Gain Amplifiers (VGA) are utilized to adjust the power excitations and the phase shifters are adopted to generate different phase parameters. The varying frequency is generated by the mixing of single LO and single DDS.

Therefore, the multichannel synchronization and multichannel consistence are determined by the modules after the power divider network, including the power divider, the phase shifter, the power amplifier, and the transmitting antennas, much fewer than transient-radiation-fields-based MSCI system, especially considering the number of active devices. For a common 6-bit digital phase shifter, the phase adjustment resolution is 5.625°, which limits the maximum phase errors. Meanwhile, even when modeling errors exist in the system, the stability of power distribution in the imaging region contributes to measurement calibrations of the radiation fields and helps to reduce the error level in field computation results. Therefore, the steady-radiation-fields-sequence-based MSCI reduces the sources of modeling errors and limits the maximum modeling errors, and makes it easier to take accurate measurements of the radiation fields, which is beneficial for robust reconstruction of the target.

### 3.2. Waveform Design and Array Configuration Optimization of the Proposed SRFS-MSCI

The diverse radiation pattern is determined by the amplitude distributions and phase distributions of the aperture fields, i.e., the amplitudes and phases of the coherent excitation signals. Hence, the waveform design is to optimize the amplitude sequence and phase sequence designs of the multiple transmitters at different frequencies. Meanwhile, recent research has indicated that random array configuration of multiple transmitters contributes to the improvement of the imaging matrix property compared with uniform array configuration [25]. Thus, the array configuration optimization should also be considered to further improve the imaging result. The radiation fields can be derived as

(13) $$E^{rad}\left(\vec{r},t\right)=\sum_{n=1}^{N}\left[\frac{F_{n}\left({\vec{r}}_{n}^{\prime },\vec{r}\right)}{4\pi |\vec{r}-{\vec{r}}_{n}^{\prime }|}rect\left(\frac{t-\frac{|\vec{r}-{\vec{r}}_{n}^{\prime }|}{c}-mT_{p}}{T}\right)A_{m,n}e^{j(\omega_{m}\left(t-\frac{|\vec{r}-{\vec{r}}_{n}^{\prime }|}{c}-mT_{p}\right)+\varphi_{m,n})}\right].$$

Therefore, the imaging matrix E is the function of $R_{3\times N}=\left[{\vec{r}}_{n}^{\prime }\right]$, $A_{M\times N}=\left[A_{m,n}\right]$, $F_{0\;M\times N}=\left[f_{m,n}\right]$ and $\Phi_{M\times N}=\left[\varphi_{m,n}\right]$, where ${\vec{r}}_{n}^{\prime }$ is the column position vector of the *n*-th transmitter, and $f_{m,n_{1}}=f_{m,n_{2}}=f_{m}$ with arbitrary $n_{1}\neq n_{2}$. When utilizing steady radiation fields sequence, the frequency matrix $F_{0}$ can be regarded as unchanged. As shown in (9) and (11), the imaging performance relies on the condition number of the imaging matrix, hence, the objective function of the waveform design and configuration optimization problem is selected as the condition number of the imaging matrix, and the abovementioned design problem is to optimize the element position configuration, amplitude sequences, and phase sequences by minimizing the objective function, expressed as

(14) $$\left[R_{opt},A_{opt},\Phi_{opt}\right]=\arg\min_{R,A,\Phi }cond\left(E(R,A,\Phi )\right).$$

To maintain the radar detecting range, the amplitudes of the multiple transmitters must be finely adjusted, for example, 1 dB power undulation can be regarded as acceptable. For digital phase shifters, the elements of phase matrix Φ are equal to discrete values. The available positions of multiple transmitting antennas have a minimum distance considering the physical size of actual antennas. Thus, the optimization problem (14) is Non-deterministic Polynomial (NP) hard. To find the global optimal solution, the Simulated Annealing (SA) algorithm [34] is utilized to optimize the position matrix R, the amplitude matrix A, and the phase matrix Φ. The parameters of SA are the temperature *T* and adjust amount J>0. The temperature *T* represents the current status of the Simulated Annealing procedure, it starts from a high temperature and gets colder when the annealing is carried out. Once the temperature reaches a minimum value, the annealing procedure is finished. The adjust amount *J* represents the total number of changing variables in a new iteration step of the SA algorithm. The minimum distance of an arbitrary pair of antennas is selected as $R_{min}$. The tuning range of the amplitude is $A_{min}\leq A\leq A_{max}$ and the available discrete phases consist of the set $\Phi_{0}=\left\{\phi_{1},\phi_{2},\ldots ,\phi_{K}\right\}$. The basic steps of SA algorithm to the phase design are listed as follows.

(1) Initial: The initial element position configuration is selected as a uniform 2D array. The elements of the initial amplitude matrix $A_{M\times N}$ are set to a same value $A_{0}$, i.e., constant power excitations. Randomly select a phase matrix $\Phi_{M\times N}$ from the available phase set $\Phi_{0}$.
(2) Set $R^{\prime }$ from R, $A^{\prime }$ from A, and $\Phi^{\prime }$ from Φ by repeating the following operations *J* times: randomly select *m* from {1,2,…,M}, *n* from {1,2,…,N}, and $A^{\prime }$ from $\left[A_{min},A_{max}\right]$, $\phi^{\prime }$ from $\Phi_{0}$, set $A_{m,n}=A^{\prime }$, $\varphi_{m,n}=\phi^{\prime }$. Change the position ${\vec{r}}_{n}^{\prime }$ of the *n*-th transmitter in the given array with the distance of arbitrary two antennas satisfying $∥{\vec{r}}_{n1}^{\prime }-{\vec{r}}_{n2}^{\prime }∥\geq R_{min},n_{1}\neq n_{2}$.
(3) Compute the imaging matrix $E(R^{\prime },A^{\prime },\Phi^{\prime })$ by (6), and calculate the objective function $cond(E\left(R^{\prime },A^{\prime },\Phi^{\prime }\right))$.
(4) Select a random value α from $\left[0,1\right]$. If $exp(-\frac{cond\left(E\left(R^{\prime },A^{\prime },\Phi^{\prime }\right)\right)-cond\left(E\left(R,A,\Phi \right)\right)}{T})>\alpha$, assign $R^{\prime }\to R$, $A^{\prime }\to A$ and $\Phi^{\prime }\to \Phi$. Else, keep the R, A, and Φ unchanged.
(5) Set (T−1)→T.
(6) If *T* equals to the ended temperature $T_{0}$, or the condition number of imaging matrix is nearly unchanged, terminate the iteration and output the optimized array configuration $R_{opt}$, amplitude matrix $A_{opt}$, and phase matrix $\Phi_{opt}$. Else, go to Step (2).

## 4. Results and Discussion

To verify the effectiveness of the proposed SRFS-MSCI method, numerical imaging experiments and a microwave anechoic chamber imaging experiment are conducted.

### 4.1. Imaging of SRFS-MSCI with Optimized Parameters

Based on the system designs shown in Figure 11 and the geometry parameters listed in Table 2, numerical imaging experiments of SRFS-MSCI are conducted. As comparison, the azimuth resolution of Real Aperture Radar satisfies

(15) $$\rho_{azimuth}=R\cdot \frac{\lambda }{D}=100\;m\cdot \frac{0.03\;m}{2\;m}=1.5\;m.$$

As shown on Section 3.2, the initial design of the proposed steady-field-sequences-based MSCI is uniform configuration, constant power excitation, and randomly selected phases.

**Figure 12:**
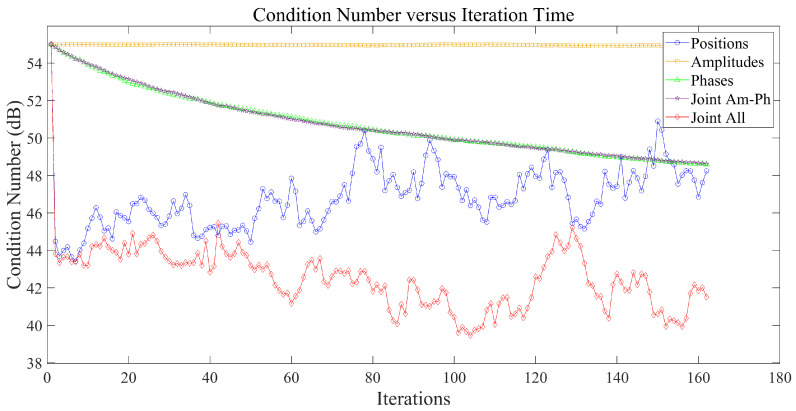
The iteration procedure of condition number versus diverse optimizing designs.

**Figure 13:**
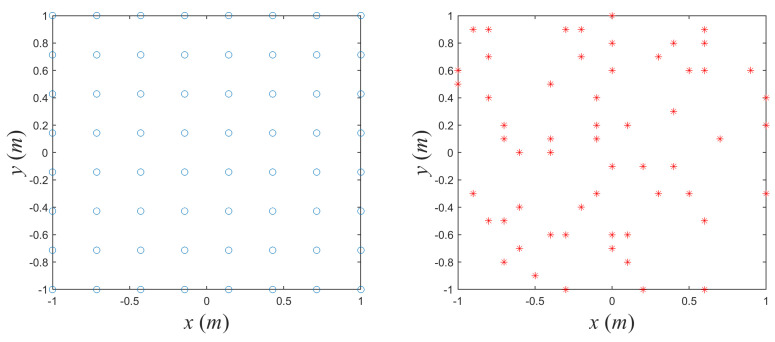
The comparison of initial uniform array configuration and optimized configuration.

The optimization of the parameters consists of 5 cases, including position optimization, amplitude optimization, phase optimization, joint amplitude-phase optimization (abbreviated as Joint AM-Ph), and all-joint optimization. The results of different iteration steps during the procedure of waveform design are illustrated in Figure 12. As can be seen, the adjustment of amplitudes within the area of ±1 dB produces tiny benefit, resulting in an almost unchanged iteration curve when utilizing joint amplitude-phase optimization. The decrease of the condition number when optimizing phase sequences represents the improving property of the imaging matrix. After several steps of the iteration, the objective function converges, meaning that an optimized design is obtained. However, the position optimization is not converged, which is possibly caused by nonunique optimal solution of the random array configuration. The array configurations are compared in Figure 13, where the optimized configuration is obtained after all-joint optimization procedure. The all-joint optimization including positions, amplitudes, and phases obtains a relatively better solution.

**Figure 14:**
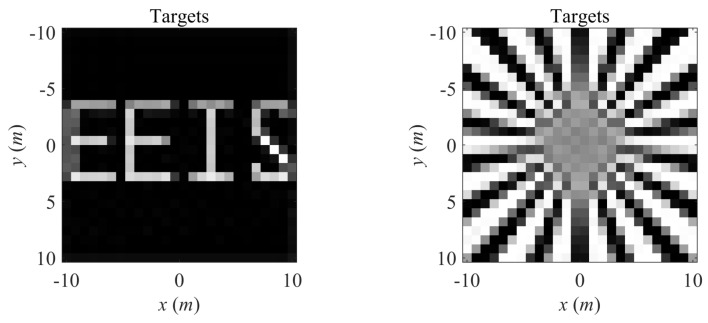
Imaging targets of SRFS-MSCI.

**Figure 15:**
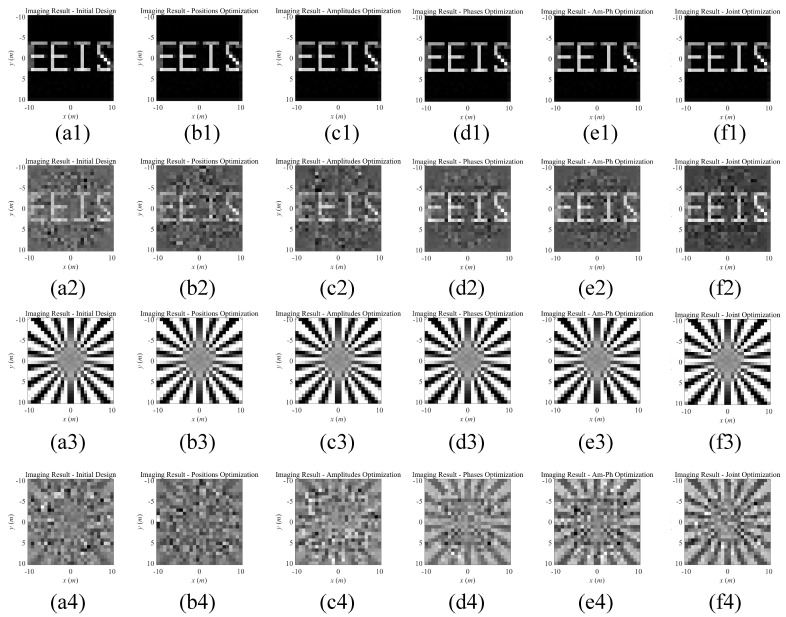
Imaging results of MSCI based on steady radiation fields sequence. (1) is results of target 1 under noise-free condition, (2) is results of target 1 under SNR= 20 dB noise, (3) and (4) are results of target 2 under noise-free condition and SNR= 20 dB noise, respectively. (**a**) represents original random designs of the parameters in SRFS-MSCI, (**b**–**d**) are results of optimized sensing matrix via changes on antenna positions, amplitudes, and phases, respectively. (**e**) is the results of joint optimization on amplitudes and phases, and (**f**) is the results of joint optimizations on all parameters.

The imaging targets are illustrated in Figure 14. With the obtained design of the radiation array, the recognizable imaging result from Truncated Singular Value Decomposition (TSVD) [35] under noise-free condition and under SNR= 20 dB are demonstrated in Figure 15. The RIE of the imaging results are listed in Table 3. The rank of the imaging matrix maintains full-rank during the above optimization process, therefore, the targets are accurately reconstructed under noise-free condition. With the diverse optimization of different parameters, improved results are obtained, as shown in Table 3. In comparison with Real Aperture Radar, the proposed SRFS-MSCI method achieves superior imaging resolution according to (15).

### 4.2. Imaging Experiment of SRFS-MSCI in Microwave Anechoic Chamber

**Figure 16:**
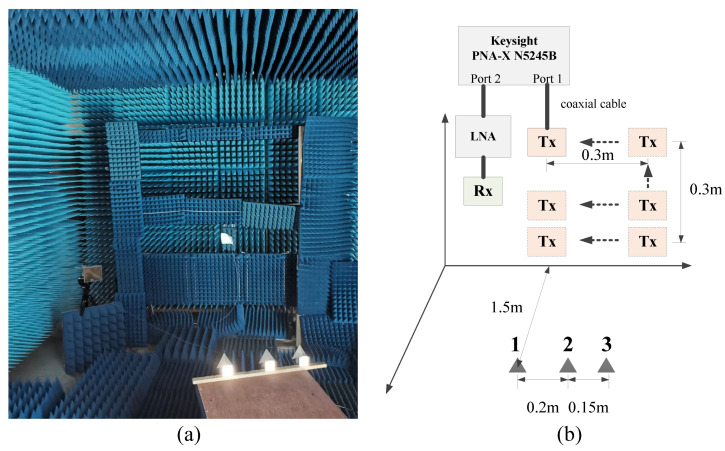
Scenario of imaging experiment of SRFS-MSCI in microwave anechoic chamber. (**a**) The real-world scenario. (**b**) The geometrical scenario diagram.

Microwave anechoic chamber imaging experiment is conducted to further verify the effectiveness of the proposed SRFS-MSCI method. The imaging scenario is demonstrated in Figure 16. Three trihedral reflectors are used as the imaging target, the distances between the three reflectors are 0.15 m and 0.2 m. The 2D range-azimuth imaging plane in size of 1 m × 1 m is discretized into 100×100 grids, with grid size equaling 1 cm × 1 cm. A single receiver is utilized in the radar system. To generate the needed steady radiation fields sequence, the system architecture illustrated in Figure 11 is approximately implemented by a time-decision scheme and digital waveform schedule [33]. A 2-D high-precision scanning antenna mount is used as the platform of a single transmitting platform. As the guide rail of the mount moves and stops at different positions, a virtual 2-D antenna array with a size of 0.3 m × 0.3 m is generated. The step interval of the transmitting antenna is 5 cm, thus, the number of effective transmitters is 49. The radar system works at X-band and the frequency of excitation signals varies from 9.5 GHz to 10.5 GHz. The Network Analyzer Keysight N5245B PNA-X is used as the Radio Frequency (RF) modules of the transmitter and the receiver, and a Low Noise Amplifier (LNA) is placed after the receiving antenna. The sweeping frequency step of the Network Analyzer is 1 MHz, thus, 1000 different frequencies are used at each transmitting position.

As demonstrated in [33], MSCI with multiple transmitters and simultaneous emitting scheme can be achieved by time-division scheme, with the predesigned radiation fields implemented by the digital waveform synthesis procedure. Therefore, the time-division scheme is adopted in the real-world imaging experiment of the proposed SRFS-MSCI method. The key modules in Figure 11 such as phase shifters, which introduces phase shift to generate the steady radiation field, are virtually achieved in the digital waveform synthesis procedure. The steady radiation fields sequence, which is generated by multiple transmitters and coherent excitations, can be implemented by the digital superposition of individual radiation fields from all transmitters. Different patterns of steady radiation fields are accomplished by diverse phase shifts in the experiment.

**Figure 17:**
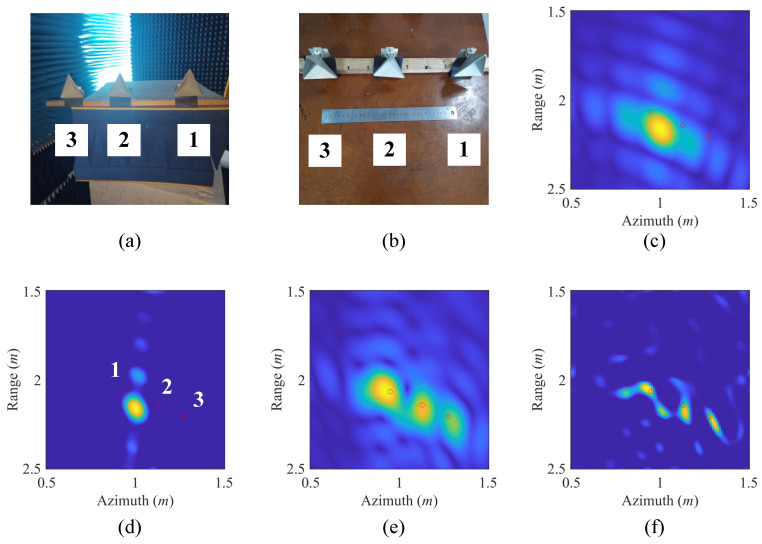
Imaging results of the real-world experiment of SRFS-MSCI. (**a**,**b**) are the photos of the three reflectors. (**c**,**d**) are the recovery results from MF and TV regularization method without phase optimization. (**e**,**f**) are the results from MF and TV regularization method with phase optimization in SRFS-MSCI.

Based on the geometric parameters of the imaging experiment, the optimization method shown in Section 3.2 is reconducted to obtain proper optimal design of the phases. Figure 17a,b show the camera images of the imaging targets. The imaging results with identical initial phases, i.e., nonoptimized phases, are obtained by Matched Filtering method (MF) [15] and Total Variation regularization method (TV) [36,37] respectively, as shown in Figure 17c,d. As a comparison, applying the phase optimization results to the digital waveform synthesis, the imaging results of the microwave anechoic chamber imaging experiment are illustrated in Figure 17e,f. The red circles in the imaging results are the true locations of the reflectors recorded by an Electronic Total Station. It should also be noted that the range and azimuth in Figure 17 are absolute coordinates measured by Electronic Total Station, not representing the relative distance between the array and target.

According to the imaging scenario, the range resolution and azimuth resolution of Real Aperture Radar can be calculated as

(16) $$\rho_{range}=\frac{c}{2B}=0.15\;m,\;\rho_{azimuth}=R\cdot \frac{\lambda }{D}=0.15\;m,$$

where $c=3\times 10^{8}$ m/s is the light speed, B=1 GHz is the frequency band, R=1.5 m is the vertical distance between radar and the target, λ=0.03 m is the wavelength, and D=0.3 m is the virtual aperture size.

As can be seen in Figure 17c,d, when the phases of the transmitters are not well designed, i.e., the steady radiation fields sequence is not achieved, the three imaging targets cannot be distinguished. After the optimization procedure, a set of optimized phases is obtained and applied to generate the feasible steady radiation fields sequence by means of digital waveform synthesis. With the utilization of steady radiation fields, three reflectors are clearly recognized and the recovered targets are located near their real positions, as shown in Figure 17e,f. The TV method achieves a better result because the gradient of the imaging targets is sparse, which meets the premise of TV regularization. The slight position mismatch between the recovered results of the reflectors and their real positions might be caused by angular glint, position measurement errors, and position shifts between visual centers and phase centers of the reflectors. Compared with range resolution and azimuth resolution of Real Aperture Radar in (16), the proposed SRFS-MSCI method achieves superior imaging results in the real-world imaging experiment.

The simulated results and the real-world imaging experiments demonstrate that, with certain designs of the transmitting parameters, the proposed SRFS-MSCI method achieves a better-behaved imaging performance with a simplified system structure than the conventional MSCI method. However, it should also be noted that, different from conventional transient-radiation-fields-based MSCI such as random frequency-hopping waveforms, the proposed SRFS-MSCI method based on coherent excitations utilizes the second-order information rather than first-order information of the radiation fields. Though the proposed SRFS-MSCI significantly reduces the system complexity and the error sources, the spatial differentiation and temporal variation of steady radiation fields sequence based on coherent excitations are definitely weaker at the same time than the transient radiation fields, thus, the random feature of TSSRF in SRFS-MSCI is not as good as in conventional MSCI, leading to a weaker imaging matrix property and worse imaging performance. Therefore, further investigations on enhancing the temporal–spatial differentiation of the radiation fields and more effective waveform optimization methods should be taken into consideration in SRFS-MSCI to obtain comparable imaging performance with transient-radiation-fields-based MSCI, while at the same time maintaining a simplified system architecture.

## 5. Conclusions

In this paper, a steady-radiation-fields-sequence-based MSCI (SRFS-MSCI) is proposed to simplify the system complexity and to remedy the defects of transient effect of the radiation fields and imaging errors that exist in the conventional MSCI system. The individual transmitters of transient-radiation-fields-based MSCI results in increasing system complexity and control difficulty, and brings in multiple types of modeling errors. The computation accuracy of transient radiation fields is sensitive to the errors, and the imaging model is hard to calibrate by the direct measurement and analysis of multiple frequencies waveform. In the proposed method, transient radiation fields are replaced by steady radiation fields sequence, which is generated from multiple transmitters excited by unique frequency coherent signals with diverse amplitude and phase information. The system architecture of the proposed method can be accomplished without multiple frequency synthesizing modules and individual controls of each transmitting channel, which significantly simplifies the system complexity and control logic of SRFS-MSCI, and reduces error sources as well. The radiation fields in the imaging region are steady within the same pulse but have diverse spatial distributions among different pulses because of the special designs of the excited signals. Hence, the proposed method possesses ability of high-resolution imaging, and measurement calibrations of radiation fields are easier to be fulfilled. To further optimize the designs of waveforms and configuration of the transmitting array, simulated annealing (SA) algorithm is applied based on the objective of minimizing condition number of the imaging matrix. Numerical imaging experiments are thus conducted, and the imaging results demonstrate the effectiveness of the proposed method. In future work, further investigations on enhancing the temporal-spatial differentiation of the radiation fields in SRFS-MSCI will be researched to achieve better imaging performance.

## Figures and Tables

**Table 1:** Simulation parameters in MSCI based on random frequency-hopping (FH) waveforms.

Simulation Parameters	Values
Transmitting array size	1 m × 1 m
Center position of transmitting array	(0 m, 0 m, 0 m)
Position of receiver	(1 m, 0 m, 0 m)
Number of transmitters	16
Array configuration	Uniform
Vertical imaging distance	100 m
Frequency band	9.5 GHz–10.5 GHz
Imaging region size	10 m × 10 m
Grid size	0.4 m

**Table 2:** Simulation Parameters.

Simulation Parameters	Values
Transmitting array size	2 m × 2 m
Center position of transmitting array	(0 m, 0 m, 0 m)
Position of receiver	(1 m, 0 m, 0 m)
Number of transmitters	64
Array configuration	Uniform
Vertical imaging distance	100 m
Frequency band	9.5 GHz–10.5 GHz
Imaging region size	20 m × 20 m
Grid size	0.8 m

**Table 3:** RIE of different designs in SRFS-MSCI.

Designs	Target1		Target2	
	Noise-Free	SNR= 20 dB	Noise-Free	SNR= 20 dB
Initial design	−240.91 dB	−3.11 dB	−233.95 dB	0.89 dB
Optimized positions	−251.87 dB	−2.91 dB	−244.19 dB	0.37 dB
Optimized amplitudes	−248.82 dB	−3.98 dB	−247.82 dB	−0.54 dB
Optimized phases	−245.41 dB	−7.95 dB	−240.91 dB	−6.73 dB
Optimized amplitudes and phases	−247.64 dB	−7.99 dB	−252.24 dB	−6.97 dB
All-joint Optimization	−263.26 dB	−8.48 dB	−262.97 dB	−9.26 dB

## Abbreviations

The following abbreviations are used in this manuscript:

MSCI	Microwave Staring Correlated Imaging
GI	Ghost Imaging
TSSRF	Temporal Spatial Stochastic Radiation Fields
CP	Correlation Process
SAR	Synthetic Aperture Radar
ISAR	Inverse Synthetic Aperture Radar
RRS	Random Radiation Source
FH	Frequency-Hopping
TSDE	Temporal-Spatial Distribution Entropy
SNR	Signal to Noise Ratio
SRFS-MSCI	Steady Radiation Fields Sequence based MSCI
SA	Simulated Annealing
RIE	Relative Imaging Error
RPE	Relative Perturbation Error
LO	Local Oscillator
DDS	Direct Digital Synthesis
